# Supplementary material for: Anemia, micronutrient deficiencies, malaria, hemoglobinopathies and malnutrition in young children and non-pregnant women in Ghana: Findings from a national survey
Source: PLoS One. 2020 Jan 30;15(1):e0228258. doi: 10.1371/journal.pone.0228258 (PMC6991996; doi:10.1371/journal.pone.0228258)
Supplement: Attachment — Submitted filename: Response to reviewers.docx [file pone.0228258.s002.docx]

**Response to reviewer’s and editor’s comments**

We thank PLOS ONE for giving us the opportunity to revise the manuscript and the reviewers for their valuable comments. We have adjusted our manuscript according to the reviewers’ and editor’s comments and hope that it now fulfills the journal’s requirements. We have included a point-by-point response to each reviewer’s and editor’s comment below.

**Reviewer’s comments**

**Reviewer 1**

This is an interesting study on prevalence of anemia, B12 and folate and micronutrient deficiencies in children and women of reproductive age in Ghana. This manuscript adds to the evidence base of women at risk of obesity.

We thank the reviewer for the encouraging words!

However, also consider the following minor and major corrections/suggestions to improve the manuscript.

- Background and discussion information: Information is missing on Ghana’s fortification program: Is there a fortification program? What type of program is it? Mandatory or voluntary? If there is a program what foods are fortified? With what nutrients in what concentrations? What evidence do we have of the quality and coverage of the program? Without this information, we cannot determine if there should be any expectation of better nutritional status in Ghana. For example, we would not expect a country without iron/folic acid fortification to have low anemia and/or serum folate deficiency. Similarly, comparing with other countries is not informative unless more information is presented on those countries’ fortification programs (What foods? What nutrients and concentrations? Quality and coverage of fortification?).

We fully agree with this point. In the introduction, we already indicated which foods are fortified with which type of micronutrients. We have however extended the introduction part on food fortification in Ghana by adding the year when fortification became mandatory for the different food types, and by being more specific on the B-vitamins added to the flour as well as mentioning some results with respect to coverage of fortified foods (lines 80-87). We have also extended the fortification part in the discussion slightly by specifically mentioning the poor adherence to the fortification level for vegetable oil and in particular for wheat flour (lines 628-630, 684).

- It is not clear if the sample size estimation has done. If yes, prevalence indicators used? Was sample size calculation based on national or regional specificity?

A paragraph on sample size calculation has been added (line 127-133)

- It is not documented the total duration of training?

The duration has been added (line 161)

- Similarly, standardization of anthropometry and phlebotomy trainings is also missing?

Details have been added (lines 164-166)

- What measures have been taken in field to avoid squeezing of finger of young children to get capillary blood sample?

The laboratory technicians were trained on the appropriate blood collection procedure during the 10-days training prior to the survey. The pricking site, removal of the first drop, the use of the second and third drops of blood for Hb and malaria testing as well as the collection into serum tubes was standardized and technicians instructed on not to squeeze the fingers of the children. During an initial intense field supervision, this aspect was particularly looked at.

**Reviewer 2**

- It wasn't clear whether this was registered at ClinicalTrials or not?

No, the trial was not registered at ClinicalTrials as it is a cross-sectional survey and not a RCT. We have however registered it with the Open Science Framework study registry as stated in the Ethics section (line 110)

- In the section of data collection procedures, it wasn't clear what methods were used to quality control of anthropometric data

We have added a sentence about this aspect (lines 201-202): scales were quality controlled on a daily basis using calibration weights.

- Line 195. Biological samples were shipped out of country. Was this information provided to study participants that their samples will sent out of the country?

There was no explicit statement in the participant information sheet about samples being exported as at time of obtaining ethical clearance, this was not required by the IRB of the Ghana Health Services. However, in the survey protocol, sample export was clearly stated with the laboratories pre-identified. And as mentioned, the protocol was made public prior to implementing the survey.

**Editor**

The points rasied by reviewer #1 are relevant and make the manuscript of greater interest to a global audience. The details on fortification, their reach and when these processes started will be of significant to neighboring countries.

As indicated in the reviewer’s 1 section we completely agree with this point and have added the relevant information in the introduction and discussion section.

**Journal Requirements:**

1. When submitting your revision, we need you to address these additional requirements.
Please ensure that your manuscript meets PLOS ONE's style requirements, including those for file naming. The PLOS ONE style templates can be found at
<http://www.journals.plos.org/plosone/s/file?id=wjVg/PLOSOne_formatting_sample_main_body.pdf> and <http://www.journals.plos.org/plosone/s/file?id=ba62/PLOSOne_formatting_sample_title_authors_affiliations.pdf>

We have adapted the manuscript to PLOS ONE’s style requirements where needed.

2. Please specify in your ethics statement: 1) whether the ethics committee approved the verbal/oral consent procedure, 2) why written consent could not be obtained, and 3) how verbal/oral consent was recorded.

The ‘Ethics statement’ section should be updated as we have added additional information on the verbal/oral consent procedure: ‘Ethical approval for the survey protocol was obtained from the Ethics Review Committee of the Ghana Health Service (GHS), number GHS-ERC-15/01/2017. The survey protocol was also registered with the Open Science Framework study registry (DOI: 10.17605/OSF.IO/J7BP9). Verbal informed consent, as approved by the Ethics Committee to be sufficient, was obtained for household interviews and was captured in the electronic questionnaire. Written informed consent was sought from eligible non-pregnant women and from the caregivers of children eligible for individual interviews, anthropometry and blood sampling.’

3. We noticed you have some minor occurrence(s) of overlapping text with the following previous publication(s), which needs to be addressed:
<http://groundworkhealth.org/wp-content/uploads/2018/06/UoG-GroundWork_2017-GHANA-MICRONUTRIENT-SURVEY_Final_180607.pdf>
<https://doi.org/10.1371/journal.pone.0155031>

In your revision ensure you cite all your sources (including your own works), and quote or rephrase any duplicated text outside the Methods section. Further consideration is dependent on these concerns being addressed.

We have rephrased some parts in the introduction, results and particularly in the discussion sections.

4. We note that you have indicated that data from this study are available upon request. PLOS only allows data to be available upon request if there are legal or ethical restrictions on sharing data publicly. For information on unacceptable data access restrictions, please see <http://journals.plos.org/plosone/s/data-availability#loc-unacceptable-data-access-restrictions>.
In your revised cover letter, please address the following prompts:
a) If there are ethical or legal restrictions on sharing a de-identified data set, please explain them in detail (e.g., data contain potentially identifying or sensitive patient information) and who has imposed them (e.g., an ethics committee). Please also provide contact information for a data access committee, ethics committee, or other institutional body to which data requests may be sent.
b) If there are no restrictions, please upload the minimal anonymized data set necessary to replicate your study findings as either Supporting Information files or to a stable, public repository and provide us with the relevant URLs, DOIs, or accession numbers. Please see <http://www.bmj.com/content/340/bmj.c181.long> for guidelines on how to de-identify and prepare clinical data for publication. For a list of acceptable repositories, please see <http://journals.plos.org/plosone/s/data-availability#loc-recommended-repositories>.
We will update your Data Availability statement on your behalf to reflect the information you provide.

We have indicated the following statement in the data availability statement in the initial submission: ‘The data underlying the results presented in the study are available from Unicef Ghana (accra@unicef.org).’ The work presented in this manuscript was conducted as part of a grant agreement with UNICEF, wherein it is stated that UNICEF and the Ministry of Health are the data owners. GroundWork and the University of Ghana were granted a limited, non-exclusive, non-transferable, world-wide, royalty-free license for the dissemination of findings in the peer-reviewed literature. Therefore, we have indicated that data are available from a third party, in this case UNICEF. We hope that this information helps clarify the situation and thank the editorial team for further guidance if required.

5. Thank you for stating the following in the Competing Interests section:

The authors have declared that no competing interests exist. The authors alone are responsible for the views expressed in this publication and they do not necessarily represent the decisions, policy or views of UNICEF.

Please confirm that this does not alter your adherence to all PLOS ONE policies on sharing data and materials, by including the following statement: "This does not alter our adherence to  PLOS ONE policies on sharing data and materials.” (as detailed online in our guide for authors <http://journals.plos.org/plosone/s/competing-interests>).  If there are restrictions on sharing of data and/or materials, please state these. Please note that we cannot proceed with consideration of your article until this information has been declared.

Please include your updated Competing Interests statement in your cover letter; we will change the online submission form on your behalf.

The updated statement is: ‘The authors have declared that no competing interests exist. The authors alone are responsible for the views expressed in this publication and they do not necessarily represent the decisions, policy or views of UNICEF. This does not alter our adherence to PLOS ONE policies on sharing data and materials.’
