# Supplementary material for: Anemia, micronutrient deficiencies, malaria, hemoglobinopathies and malnutrition in young children and non-pregnant women in Ghana: Findings from a national survey
Source: PLoS One. 2020 Jan 30;15(1):e0228258. doi: 10.1371/journal.pone.0228258 (PMC6991996; doi:10.1371/journal.pone.0228258)
Supplement: Attachment — Submitted filename: Response to Reviewers.docx [file pone.0228258.s003.docx]

**Response to editor’s comments**

We thank PLOS ONE for re-assigning our manuscript to a new editor and appreciate the additional points raised by the new editor. We will give a point-by-point response to the additional editor’s comments below. We are glad to see that we have addressed all comments of the two initial reviewers.

**Editor’s comments**

Line 71-73: the claim that “no national assessment of the prevalence of the haemoglobin disorders sickle cell disease and trait or thalassemia has previously been conducted” is wrong. At least I know that the Malawi Micronutrient Survey 2016 measured these disorders at national level. I recommend you to correct this claim and compare your findings with the finding of the aforementioned survey.

In this part of the manuscript we are specifically referring to Ghana and therefore say that no national data is available. We have added ‘in Ghana’ at the end of the sentence to make this clear (line 76). There have been nationally representative assessments of hemoglobin disorders in other countries, so we hope to have clarified sufficiently that we are specifically referring to Ghana.

In the background section, please add a paragraph that concisely present the micronutrient situation in the study country.

We have added prevalence data for those micronutrient deficiencies for which data is available in the literature (lines 70-74), which however relies on only two publications and is not national. The scarcity of the publicly available and nationally representative data was the main justification for doing the study.

Line 235-36: “For both PSC and WRA, ferritin concentrations were adjusted for elevated AGP and CRP according to the procedure recommended by Thurnham”. Can you please present both adjusted and non-adjusted prevalence figures in the manuscript?

We prefer not to present the unadjusted prevalence figures for both, iron deficiency as well as vitamin A deficiency, as we strongly support the adjustment for inflammation in an area such as Ghana with a high infection burden. We have however added the unadjusted ferritin and RBP concentrations in the footnote of Tables 2 and 4 to give an idea on how different the adjusted values are from the unadjusted ones.

Line 315-16: please provide the definitions for “minimum acceptable diet”, “continued breastfeeding at 1 year” etc in the manuscript.

In the method section (lines 274-276) we are referring to the guidelines used for calculating the minimum acceptable diet as well as other IYCF indicators. As these are standard IYCF indicators commonly used and the focus of this paper is not on IYCF, we prefer not to give the definitions for all indicators in this manuscript to keep the manuscript focused on the biomarker topic.

Table 1: among many core and optional IYCF indicators it is not clear how the authors selected and presented only two of the indicators I mentioned above. I recommend them to present all the core indicators applicable to children 6-23 months in the table.

We agree and have included all IYCF indicators that we have assessed in the survey in Table 1 and have done some changes in the text (lines 344-345). The initial decision to only present a few selected indicators was made based on the fact that the focus of this manuscript is on biomarkers.

It is important that the serum ferritin concentration had been adjusted for inflammation. But it is not clear why inflammation adjustments had not been made for retinol and retinol binding protein. Can you discuss the matter further?

This is a very good point, thank you for spotting this. We have missed to state in the method section that we indeed also adjusted RBP for inflammation using Thurnham. This is now added in line 258. For both, the RBP concentration and VAD prevalence data stated in Tables 2 and 4, we used Thurnham adjusted values. This was actually stated in the footnote of Table 4 and we have added a footnote to Table 2 as well. We have additionally added a footnote in relation to iron status to also indicate that ferritin concentrations were adjusted for inflammation in both tables.
