# Supplementary material for: Anemia, micronutrient deficiencies, malaria, hemoglobinopathies and malnutrition in young children and non-pregnant women in Ghana: Findings from a national survey
Source: PLoS One. 2020 Jan 30;15(1):e0228258. doi: 10.1371/journal.pone.0228258 (PMC6991996; doi:10.1371/journal.pone.0228258)
Supplement: Attachment — Submitted filename: Reviewer Comments.docx [file pone.0228258.s001.docx]

This is an interesting study on prevalence of anemia, B12 and folate and micronutrient deficiencies in children and women of reproductive age in Ghana. This manuscript adds to the evidence base of women at risk of obesity.

However, also consider the following minor and major corrections/suggestions to improve the manuscript.

- Background and discussion information: Information is missing on Ghana’s fortification program: Is there a fortification program? What type of program is it? Mandatory or voluntary? If there is a program what foods are fortified? With what nutrients in what concentrations? What evidence do we have of the quality and coverage of the program? Without this information, we cannot determine if there should be any expectation of better nutritional status in Ghana. For example, we would not expect a country without iron/folic acid fortification to have low anemia and/or serum folate deficiency. Similarly, comparing with other countries is not informative unless more information is presented on those countries’ fortification programs (What foods? What nutrients and concentrations? Quality and coverage of fortification?).

- It is not clear if the sample size estimation has done. If yes, prevalence indicators used? Was sample size calculation based on national or regional specificity?
- It is not documented the total duration of training?
- Similarly, standardization of anthropometry and phlebotomy trainings is also missing?
- What measures have been taken in field to avoid squeezing of finger of young children to get capillary blood sample?
